# Laser interstitial thermotherapy (LITT) for the treatment of tumors of the brain and spine: a brief review

**DOI:** 10.1007/s11060-020-03652-z

**Published:** 2021-02-21

**Authors:** Clark Chen, Ian Lee, Claudio Tatsui, Theresa Elder, Andrew E. Sloan

**Affiliations:** 1 University of Minnesotta, Minneapolis, USA; 2 Henry Ford Hospitals, Detroit, USA; 3 MD Anderson Cancer Center, Houston, USA; 4 Seidman Cancer Center, University Hospitals, Shaker Heights, USA; 5 Case Comprehensive Cancer Center, Cleveland, USA

**Keywords:** LITT, Laser interstitial thermotherapy, SLA, Stereotactic laser ablation, Glioma, Brain metastasis, Radiation necrosis, Meningioma, Spinal metastasis

## Abstract

**Introduction:**

Laser Interstitial Thermotherapy (LITT; also known as Stereotactic Laser Ablation or SLA), is a minimally invasive treatment modality that has recently gained prominence in the treatment of malignant primary and metastatic brain tumors and radiation necrosis and studies for treatment of spinal metastasis has recently been reported.

**Methods:**

Here we provide a brief literature review of the various contemporary uses for LITT and their reported outcomes.

**Results:**

Historically, the primary indication for LITT has been for the treatment of recurrent glioblastoma (GBM). However, indications have continued to expand and now include gliomas of different grades, brain metastasis (BM), radiation necrosis (RN), other types of brain tumors as well as spine metastasis. LITT is emerging as a safe, reliable, minimally invasive clinical approach, particularly for deep seated, focal malignant brain tumors and radiation necrosis. The role of LITT for treatment of other types of tumors of the brain and for spine tumors appears to be evolving at a small number of centers. While the technology appears to be safe and increasingly utilized, there have been few prospective clinical trials and most published studies combine different pathologies in the same report.

**Conclusion:**

Well-designed prospective trials will be required to firmly establish the role of LITT in the treatment of lesions of the brain and spine.

## Introduction

Laser Interstitial Thermotherapy (LITT; also known as Stereotactic Laser Ablation or SLA) is a minimally invasive treatment modality that has recently gained prominence in the treatment of malignant brain tumors [1–15]. Historically, the primary indication for LITT has been for the treatment of recurrent glioblastoma (GBM). However, indications have continued to expand and now include gliomas of different grades, brain metastasis (BM), radiation necrosis (RN) as well as spine tumors [1–14, 16, 17]. There are also small numbers of other tumor types which have been treated with LITT. This paper serves as a brief review of these topics with conclusions in each section.

Thermal ablation using cryoablation, radiofrequency, ultrasound and laser has a long tradition of efficacy in many tumor types including brain tumors [15, 18, 19]. However, while laser has long been utilized for open surgery, the earliest cases of LITT for brain tumors were reported in 1983 [20]. The efficacy of early LITT was highly variable, performed with different, unique platforms at a small number of institutions, and thus limited to sites with clinical interest and technological support [12, 21–23]. After 2006, three technological innovations led to commercialization of LITT which in turn led to increased adoption for tumors and epilepsy: the development of MRI compatible, cooled probes; reproducible MRI thermometry; and software that could integrate the findings of repeated thermometry to identify cumulative thermal damage of several individual LITT treatments. The two currently commercially available LITT platforms are the “Visualase” system approved by the FDA in 2009 after a phase I study in 4 patients with brain metastasis, and the Monteris NeuroBlate Platform, which was first approved by the FDA in 2013 after a first in man trial in 10 patients with unresectable recurrent glioblastomas [11, 24]. The features of these platforms have been described previously [25–27].

### Treatment of low grade gliomas (WHO grade I-II)

Eighteen studies and case reports have described treatment of 98 patients undergoing LITT therapy for low grade gliomas (Table 1) [21, 28–38]. The majority of tumors were less than 35 mm in diameter and were considered unresectable because of location within eloquent brain or due to a perceived high risk of conventional open surgery. LITT was usually used as the primary mode of treatment and not followed by adjuvant therapy. In the overwhelming majority, tumors were in areas of eloquence or very high surgical risk [29, 31, 33, 34, 39–42]. In general, the use of LITT in treating low grade (WHO I–II) glioma appears to be well tolerated without permanent neurological deficits. The majority of tumors were stable, or demonstrated partial response. One study provided mean values for time to progression and survival of 16 and 34 months, respectively [21]. No prospective clinical trials have been reported.

**Table 1:** Analysis of low grade glioma patients treated with LITT

Study	Tumor grade (Number of patients)	Patientdemographics (M/F, age range)	Inoperable due to tumor location? (yes/no)	*De* *novo* or *follow on *LITT	Intra operative MRI guidance (yes/no)	Adjuvant postoperative therapy? (yes/no)	Clinical results
Ascher [28]	II (2)	1M/1F, 8 and 35	Yes	De novo	Yes	N/A	Male pt at 4 years out showed no presence of tumor
Roux [29]	I (2) II (2)	2M/2F, 10–20	Yes	De novo	No	No	No perioperative morbidity/mortality
Kahn [30]	II (3)	2M/1F, 29–68	Yes	De novo (1 follow on)	Yes	2 no/1 yes	3 months post-op LITT, lesion size decreased 15–87% (avg. 51%)
Kahn [31]	II (2)	1M/1,: 40 and 27	Yes	De novo	Yes	No	No peri-procedural side effects noted
Schwabe [32]	II (11)	7M/4F, 23–68	Yes (8/11)	De novo	No	No	Total lesion size decreased by 50% within 90 days in all patients. 7/18 pts had recurrence outside laser field (unknown f/up)
Leonardi [21]	II (7)	Not specified, mean age: 46.9	Yes	De novo (6/7)	Yes	No	Mean survival time: 34 months Mean time to progression: 16 months
Von Tempelhoff [33]	II (2)	1M/1F, 30 and 40	Yes	De novo	Yes	No	No clinical or radiological recurrence at 28 month follow up
Jethwa [86]	I–II (3)	N/A, 13–58	No	Follow on	Yes	No	Majority of patients discharged on post-operative day 1
Patel NV [89]	I–II (4)	2M/2F, 13–59	Yes	De novo (2 follow on)	Yes	Fractionated radiation in 1 patient	Average recurrence time 4.4 months. 1 patient remained recurrence-free at 40 months
Patel NV [62]	I–II (7)	N/A, 10–82	Unclear	Follow on	Yes	No	No peri-procedural morbidity
Buckley [37]	I (3)	2M/1F, 7–18	Yes	De novo (1 follow on)	Yes	Adjuvant everolimus in 2 patients	Durable clinical and radiographic tumor control in 2 of 3 cases
Dadey [38]	I (2)	2F, 13 and 14	No	De novo	Yes	No	Tumor shrinkage on 3-month MRI, stable clinically
Dadey [87]	I (2)	1M/1F, 36 and 55	Yes	De novo	Yes	No	1 patient experienced post-op ophthalmoplegia and dysarthria, which resolved to neurologic baseline at 6 month follow up
Tovar-Spinosa [85]	I–II (11)	6M/5F, 4–17	Unclear	6 De novo, 5 follow on	Yes	Adjuvant Everolimus in 1 patient	Only 1 patient showed signs of progression and was started on Everolimus
Patel [34]	I–II (23)	Not specified	Unclear	Follow on	Yes	No	13.7% of patients had a post-op neurologic deficit; 64.3% of these had complete resolution of deficit at 1 month follow up
Miller [35]	I–II (6)	Not specified, 10–17	Unclear	De novo	Yes	Adjuvant chemotherapy in 1 patient	All patients discharged on post-operative ay 0 or 1. Four patients demonstrated no recurrence on follow up to date
Rennert [5]	I–II (5)	Not specified, mean age: 50.7	Yes (46% of lesions treated)	De novo	Yes	N/A	No perioperative morbidity
Karsy [36]	I (1)	1M, 5	No	De novo	Yes	No	No perioperative morbidity
Total	I–II (98)	Age range: 5–82					**Well tolerated overall**

### High grade gliomas (WHO grade III-IV)- including recurrent GBMs

Nineteen studies highlighted experience with LITT of high-grade gliomas (WHO grade III and IV), reporting 252 cases (Table 2) [9, 21, 22, 24, 25, 27, 30, 32, 33, 43–46]. The age of patients varied from 24 to 78 years. All treated tumors were < 50 mm in diameter. LITT was mainly utilized for management of residual or recurrent neoplasms, when other treatment options had been exhausted. In general, thermal therapy was well tolerated. The most common complications were seizures as well as complications typical of this population of patients with malignant brain tumors and limited mobility including deep venous thrombosis, and pulmonary embolism. These complications were encountered, on average, in 3.5%, 4.7% and 2.4% of cases, respectively, with slightly higher risk in patients with recurrent tumors [21, 24, 33, 44–46]. Moderate perilesional brain edema after surgery was common [24, 32, 43, 46]. Transient postoperative neurological decline was occasionally noted, whereas permanent deficit was only encountered in 0–10% of patients (in average, 4.8%) [22, 25, 33, 47]. The risk of deficit was associated with early use of the technology, as well as treatment of large and deep seated or eloquent lesions.

**Table 2:** LITT for high grade glioma

Study	Tumor grade (Number of patients)	Patientdemographics (M/F, age range)	Inoperable due to tumor location? (yes/no)	*De* *novo *or *follow on* LITT	Intraoperative MRI guidance (yes/no)	Adjuvant postoperative therapy? (yes/no)	Clinical results
Sakai [43]	III–IV (3)	Not specified, 37–56	Yes	De novo	No	No	1 pt died at 23 months due to recurrence, 2 pts’s tumor disappeared at 12 to 34 months follow ups
Kahn [30]	III–IV (3)	1M/2F, 51–68	Unclear	Follow on	Yes	No	1 pt died of heart failure; in grade III pt no sign of recurrence at 9 months, in grade III-IV tumor recurred at 2 months
Schwabe [32]	III–IV (3)	2M/1F, 48–63	Unclear	De novo	No	No	Total lesion size decreased by 50% within 90 days in all patients. Increase in radiologic perifocal edema (maximum extent at 4–27 days); subsided after mean of 23 days
Reimer [44]	III–IV (4)	2M/2F, 36–62	Yes	Follow on	Yes	No	Pt. 1 with no sign of tumor recurrence at 6 months; pts 2 and 3 had tumor recurrence at 6–8 months, pt 4 had no recurrence at 12 months
Sneed [45]	IV (35)	Not specifiedd, 24–73	Unclear	35 follow-on treatments with LITT post surgery and brachytherapy	Yes	25 of 35 underwent reoperation	Time to progression of glioblastoma: 49 weeks with LITT. Survival longer with LITT: 80 weeks versus 76 weeks without LITT (log rank; *p* = 0.04)
Leonardi [21]	III–IV (17)	Not specified, mean age 55	Unclear	Follow on	Yes	No	GRADE III Pts after LITT: MST 30 m, MPT 10 m, grade IV pts after LITT: MST 9 m, MPT 4 m
Von Tempelhoff [33]	IV (2)	2M, 59–68	1st yes, 2nd no	Follow on treatment in patients refractory to other therapies	Yes	Yes (oral chemotherapy –temozolomide	Good to complete tumor control 7 to 13 months post LITT
Schwarzmaier [22]	rec IV (16)	10M/6F 44–69	Yes	Follow on treatment in nonsurgical candidates	Yes	Yes	MST: 11.2 ± 2 mths LOS shorter with LITT than with open resection. Survival time using LITT longer than SOC/palliative care (< 5 months.)
Carpentier [27]	Rec IV, (4)	3M/1F, 40–58	Yes	Follow on (prior resection/ chemo/ radiation	Yes	No	MOS after LITT: 10.5 months. OS after LITT: 11 months
Jethwa [86]	III–IV (7)	Not specified, 9–84	Yes (3 patients)	Follow on	Yes	No	1 patient had post-operative edema requiring surgery
Sloan [24]	IV (10)	8M/2F, 34–69	Yes (8 Patients)	Follow on; patient refractory to other treatments	Yes	No	Median ST of 316 days
Hawasli [25]	IV, anaplastic oligodendroglioma (11)	8M/3F, 34–78	6 Patients with deep lesions, 1 with Corpus Callosum tumor	6 De novo; 3 with prior craniotomy, radiation therapy and chemo; 1 with radiation and chemo only	Yes	Yes	Preliminary overall median progression-free survival and survivalfrom LITT were 7.6 and 10.9 months, respectively
Patel [89]	III–IV (3)	Not specified, 10–82	Unclear	Follow on	Yes	No	No peri-procedural morbidity or mortality
Mohammadi [9]	III (10) IV (24)	21M/13F, 19–79	Yes (8 deep lesions, 1 with corpus callosum tumor)	49% de novo, 51% follow on	Yes	Adjuvant chemotherapy and/or radiation in 14 patients (42%)	Median progression-free survival 5.1 months
Pisipati [40]	IV (5)	Not specified	Yes	De novo	Yes	Adjuvant chemotherapy and radiation in 3 patients	Resection following LITT did not increase the length of hospital stay except in one patient. No new neurologic deficits identified
Thomas [41]	IV (24)	Not specified, mean age 52.4	Yes	Follow on	Yes	Adjuvant chemotherapy in 16 patients, 7 of which also received radiation therapy (newly diagnosed GBM)	Median overall survival 8 months in newly diagnosed GBM, > 7 months in recurrent GBM
Patel [34]	IV (24)	Not specified	Unclear	Follow on	Yes	No	13.7% of patients had a post-op neurologic deficit; 64.3% of these had complete resolution of deficit at 1 month follow up
Laurent [42]	III–IV (9)	5M/4F, 49–80	Unclear	Follow on	Yes	1 patient	No major complications. 30 day re-admission rate and mortality of 0
Rennert [5]	III–IV (38)	Not specified, mean age: 50.7	Yes (46% of lesions treated)	De novo	Yes	N/A	No perioperative morbidity
Total	III–IV (252)	Age range: 9–84					

Length of hospital stay after LITT for recurrent GBM (rGBM) was shorter in comparison to tumor resection [48, 49]. During follow-up, tumors usually demonstrated volume reduction [24, 32, 33, 43]. Several clinical series also demonstrated extended survival (median, 9.0–11.2 months) after LITT in patients with rGBM refractory to other treatment and not suitable for re-resection, which was beneficial in comparison to best palliative care [21, 22, 24, 25, 27, 46].

Another challenge has been tumor volume. Most of the lesions treated in the literature, and the majority of the patients with the best outcomes, are those with volumes of less than 10 cm^3^ (which corresponds to a radius of 1.33 cm) [1, 5]. This has been attributed to the observation that larger tumors typically cause more swelling which is poorly tolerated. Wright et al. demonstrated that LITT can be combined with a mini-craniotomy and trans-sulcal, trans-tubular approach which addresses the challenge of post-treatment swelling in patients with large, difficult to access large [1]. The authors noted that in this subgroup of patients, minimally invasive LITT combined with a small craniotomy using a tubular retractor system facilitated a radical resection required to achieve survival advantage in such patients without the need for a larger craniotomy. They noted that LITT changed the consistency of the tumor, devascularizing it and making it more “suckable” and thus more easily and safely removable. Median survival in this study of 10 patients with median age of 65 was more than 16 months, with PFS of 9.3 months [1]. Another group demonstrated that LITT induces a transient breakdown of the peritumoral blood-tumor barrier which has potential to improve drug delivery to intracranial tumors [50]. Several trials assessing the utility of LITT to augment drug delivery have been proposed and some are underway.

The two largest case series of LITT for GBM also demonstrated a longer survival time in patients using LITT when no other therapeutic options were available to the patient except for best palliative care. The first in man trial of LITT for inoperable rGBM demonstrated a median survival of 10.5 months after LITT compared to the expected 3–5 month median survival with best conventional care (n = 10 patients) which was replicated in another series of four cases [24, 27]. However, it should also be noted that recurrent GBM amenable to treatment with LITT are highly selective subpopulation of GBM with unifocal tumors less than 5 cm in diameter, and thus not necessarily generalizable to many historical controls. In general, LITT has been well tolerated although temporary and permanent injuries have also been observed after LITT. Two studies, totaling 32 patients, have examined the length of hospital stay (LOS) in LITT versus open surgical resection scenarios, and both proved a shorter length in favor of LITT [22, 25]. While only three of the published studies were prospective clinical trials, three ongoing studies examine the potential of LITT to potentiate chemotherapy or immunotherapy [11, 14, 24, 51–53].

## Conclusions

LITT has been in development for the past two decades and has now demonstrated efficacy in the treatment of glioma of various grades and types. While most of the publications have been retrospective case studies, these demonstrate that LITT is a potentially beneficial focal form of therapy in patients with gliomas who are not otherwise candidates for open surgery or have exhausted other therapeutic modalities. This noninvasive, anatomically and physiologically personalized form of treatment has the added asset of incurring substantially less hospitals costs and posing much less peri-procedural medical risks to the patient.

### *Treatment of brain metastasis*

An estimated 25–35% of all cancer patients suffer from brain metastasis (BM), though the true incidence of BM remains unknown and is expected to rise in parallel with the increasing incidence of cancer and with the aging of society [54]. Additionally, since most effective cytotoxic drugs and monoclonal antibodies drugs penetrate the blood brain barrier (BBB) only poorly, patients are living longer from diagnosis until tumors develop in the CNS and possibly other sequestered locations.

The lack of efficacy of chemotherapy for BM treatment has resulted in a dependence on surgical resection and/or ionizing radiation. Radiation can be delivered as whole brain radiation therapy (WBRT), which is dosed to the entire brain typically over 10–15 sessions; or as stereotactic radiosurgery (SRS), where multiple high energy beams converge specifically on the target with rapid dose falloff in 1–5 fractions. Because WBRT is associated with increased risk of neuro-cognitive deficits, the majority of patients afflicted with a limited number of BM are treated with SRS [14, 55–57]. The current clinical practice suggest that SRS remains the first line treatment for BM patients in this context.

In recent years, however, LITT, has emerged as a therapeutic option for BM that recur after SRS [3, 5]. These BM will be referred to as BMRS (brain metastasis recurred after SRS). In this section, the efficacy of LITT in this context will be reviewed. Focus will lie only on studies where histologically confirmed BM recurrence were treated with LITT. Studies primarily focusing on radiation necrosis (RN) will be reviewed in a separate section.

#### Local control

Carpentier et al. were the first to report local control for LITT treated BMRS [58]. In this study, local control was defined as post-treatment contrast-enhancing volume (CEv) smaller than pre-treatment CEv. Using this definition, the authors report that local control was 60% for partially ablated lesions (n = 6) and 85% for completely ablated lesions (n = 9). Ahluwalia et al. reported a multi-center study involving 42 patients (20 with BMRS) treated with LITT [57]. Of the BMRS patients, local control was observed in 100% of completely ablated BMRS, but only 37.5% of incompletely treated lesions suffered disease progression. Similarly, Ali et al. found that 100% local control was achieved with completely ablated BMRS, but only 65.3% if < 80% of BMRS was ablated [14]. In a subset of these patients treated with adjuvant radiosurgery, local control was 100% irrespective of the extent of ablation. Rao et al. similarly reported local control in 85.7% of patients undergoing complete ablation a series of 14 BMRS patients [59].

#### Overall survival

Median overall survival in LITT-treated BMRS patients ranges from 5.8 months to 19.8 months, with one year survival of 0–65% [25, 58–60]. The longest reported survivorship after LITT was 30 months. Most patients died within months of laser ablation, suggesting that systemic disease control remains a key determinant of survival for patients with BMRS as with SRS [1, 25, 58–60].

#### Neurologic and functional status

Chaunzwa et al. report stable or improved KPS was observed in 13/22 patients (59%) who had pre-operative KPS of 70–100. Ablation of > 90% of BMRS was associated with a higher likelihood of improved KPS. This study did not stratify results by BMRS or RN and should be interpreted in this context [61]. Similarly, in a series of 42 BMRS patients, Ahluwalia et al. noted 87% of patients experienced stable or improved KPS at last follow-up [57]. Reduction in steroid requirement was reported in 31% of these cases.

#### Complications

The complication rate associated with LITT of BMRS was < 10%. Complications include catheter misplacement, infection, hydrocephalus, hemorrhage, thermal injury to normal brain, and malignant edema requiring re-operation for hemicraniectomy. In the four cases of post-LITT malignant cerebral edema requiring hemicraniectomy, ablated volume ranged from 29 to 70 cm^3^ [14, 62–64]. This suggests that LITT of larger lesions should be avoided, or combined with debulking as previously noted [1].

#### Conclusion

The available retrospective studies suggest that LITT is a good option for patients suffering from BMRS. Neurological outcome is generally favorable and complete ablation increase the likelihood of local control. Prospective studies will be needed to better understand optimal application of LITT for BMRS.

### LITT for cerebral radiation necrosis

Radiation necrosis (RN), a severe local tissue reaction which most commonly occurs 3–12 months after completion of radiation therapy, is a common complication of radiation therapy (RT) for primary and metastatic brain tumors, occurring 4.7–9.2% in patients with metastatic brain tumors undergoing SRS with higher doses associated with a higher risk of RN [65–68]. Although most cases of RN are self-limiting, symptomatic lesions may require treatment. Steroids are the mainstay of treatment for RN by inhibiting the pro-inflammatory cytokine response from radiation [69]. However, the treatment response is transient and some patient may become steroid dependent. In addition, steroid treatment carries significant side effects, including infection, gastrointestinal bleeding, myopathy and diabetes. Bevacizumab, a vascular endothelial growth factor inhibitor, has also been shown to be effective in RN [70]. Other treatment such as Vitamin E, hyperbaric oxygen, pentoxifylline and anticoagulation have also been used with limited efficacy [71–73]. For a minority of patients, surgery may be necessary to relieve mass effect. Open surgery provides the benefit of immediate relief of mass effect and tissue diagnosis, but carries attendant risks of surgical morbidity, including stroke, hemorrhage, and wound-related complications and usually requires a pause in systemic treatments to allow for wound healing.

LITT has emerged as an increasingly popular treatment for recurrent or enlarging enhancing lesions after RT and or SRS for brain tumors. When performed with a stereotactic biopsy, as it is typically performed, LITT provides the advantage of combining the diagnostic procedure (to assess tumor recurrence versus RN) along with cytoreductive treatment with a minimally invasive technique approach and short hospitals stay. As such, it may minimize the amount of time needed to be off systemic treatments and lessen recovery time. On the downside, it is not suitable for patients already suffering from mass effect and is subject to sampling error as with all stereotactic biopsies. No RCTs have been performed to date examining the role of LITT in RN, but multiple retrospective studies have demonstrated LITT to be a promising treatment modality.

The use of LITT for RN was first described in 2012 [74]. Since then, multiple retrospective studies have been published examining the role of LITT in RN [11, 57, 59, 60, 75–81]. Most studies have contained a mixture of patients with either recurrent tumor or RN post-SRS for brain metastasis or glioma, with three focused solely on RN [11, 57, 59, 60, 75–81].

### Control of RN and complications

In the studies where the ablation percentage was calculated, between 86.4 and 100% of the contrast enhancing volume was ablated [57, 61, 75, 77, 80, 82]. Early studies were encouraging, with most patients achieving palliation of symptoms, especially in those patients who were able to attain complete ablations [11, 79]. Hong et al. performed a retrospective cohort study comparing LITT to craniotomy for patients with progressive enhancement post-SRS for metastatic disease. For the patients undergoing LITT with biopsy proven RN, they demonstrated a local control rate of 87.4% at 18 months. Of note, in patients who were found to have tumor, local control rate was 61.5% compared to 87.4% for those with RN [75]. Similarly, Ahluwalia et al. showed a 91% local control rate at 12 weeks for the 19 patients with RN who underwent LITT, compared to 54% for the 20 with recurrent tumor [57]. Smith et al. published a case series with a mixture of histologies including low and high grade gliomas, meningioma and metastatic disease. For the seven patients with metastatic disease, the local control rate was 86%. Several other studies were done without biopsies, thus response rates represent a mixture of recurrent metastatic disease and RN [11, 59, 76]. Hernandez et al. reported a series of 59 patients with 74 treated lesions, which was a continuation of an earlier work by Rao et al. [76]. In that study, only ten of the 59 (16.9%) patients had local recurrence of disease after LITT.

Complication rates ranged from 0% -30% [11, 57, 59, 60, 75–81]. Most of the complications were due to hemorrhage, seizures, new or worsened motor deficit due to proximity of the corticospinal tract, as well as systemic complications such as myocardial infarction, deep vein thrombosis and pulmonary embolism. This is in line with other LITT case series. Of note, in the retrospective cohort study performed by Hong et al., they found a similar rate of complications in patients undergoing LITT vs. craniotomy [75].

### Weaning of steroids

RN can lead to steroid dependence which is sometimes as debilitating as recurrent tumor, thus, most of the studies examined the ability to wean off steroids after LITT [11, 57, 59, 61, 75, 78–80, 82]. Due to variability in reporting, it is difficult to draw generalized conclusions. However, typically steroids could be weaned within 2–4 weeks in many cases (70–100%) [59, 61, 77, 78]. In two of the larger series by Ahluwalia et al. and Hong et al., 31% were weaned off at 12 weeks and 34.8% patients were weaned off at 4 weeks, respectively [57, 75]. Finally, in a multicenter retrospective case series by Chaunzwa et al., 19/30 (63.3%) patients had adequate steroid use data for analysis. Eleven patients had symptom relief with steroids pre-LITT (36.7%), of which 9 (30%) were able to wean off steroids completely by a median of 5 weeks [61].

## Conclusions

LITT appears to be a viable treatment for RN in several retrospective studies, though no prospective studies or trials have been reported. Complication rates for LITT in the setting of RN are similar to LITT performed for other oncological conditions and appear to be lower than the rate seen in craniotomies. Most patients with biopsy proven RN will be able to wean off steroids. Finally, biopsy does appear to confer valuable data, as it may influence treatment post-LITT.

### LITT for other neoplastic conditions

The bulk of the reported data about LITT for oncology indications has focused on gliomas, recurrent metastases and RN. However, though there have been no prospective studies of other types of intracranial lesions, there have been a few case series reviewing the use of LITT for dural-based lesions, largely meningiomas [82–84]. Ivan et al. described a group of five patients with recurrent extra-axial masses including three grade I meningiomas, one grade III meningioma, and one solitary fibrous tumor. All had clear evidence of tumor progression after initial treatment including craniotomy and radiation, but were poor candidates for open surgery. In this case series, the grade I meningiomas showed good response to LITT, with 52% reduction of the size of the lesions at 3 months. The other two patients showed early progression at three months for the anaplastic meningioma and ten months for the solitary fibrous tumor, in line with the aggressive pathology of the masses [84]. A follow up study of the same group of patients demonstrated no evidence of recurrence of the grade I meningiomas with a follow up of seven to ten months [84]. Similarly, in another small retrospective case series, two patients with anaplastic meningiomas and one with benign meningioma were treated with LITT. Average ablation coverage was 75%, with both of the anaplastic meningioma patients having early progression [82]. The grade 1 meningioma was recurrence-free at 28 weeks. Unfortunately, this patient had severe edema after the procedure leading to near hemiplegia. Although the hemiplegia eventually resolved, it took six months to do so [82]. Although these studies describe only a very small number of patients, available data demonstrate that the localized pathology of the meningiomas defines the localized clinical course of this disease, which tends to recur locally rather than at distant sites regardless of what treatment modality is utilized.

LITT has also been employed for other more commonly seen pediatric intra-axial pathologies, such as ependymomas, primitive neuroectodermal tumors, subependymal giant cell astrocytoma, pilocytic astrocytoma, medulloblastoma, choroid plexus xanthogranuloma, and ganglioglioma [62, 85–88]. All are relatively small case series, but treatment results and complications are in line with the results seen in adult patients [62, 85–89]. Notably in the case series by Tovar-Spinoza and Choi, many of the lesions treated were difficult or potentially dangerous areas to treat such as the cerebellar peduncle, thalamus or midbrain. Despite this, only two out of eleven patients (18.2%) had complications consisting of transient weakness in both patients, and akinetic mutism and eye motion disorder in one of the patients, of which both patients largely recovered. In addition, durable response to treatment was seen, with decrease in tumor volume out to six months [85]. In summary, LITT for pediatric pathologies is analogous to adult tumors with the caveat that the pediatric patients may have greater capacity for neurological recovery.

### *Treatment for spine tumors*

Advances in cancer therapy has led to longer survival time of patients with various cancer subtypes. Multidisciplinary teams of spine surgeon, radiation and medical oncologists, pain and rehabilitation specialists, and interventional radiologist have formed in order to deliver the best spine cancer treatment. The goals of treatment for metastatic spine disease remain palliative and aside from traditional goals such as local tumor control, achievement of symptom palliation and improved health-related quality of life (HRQoL) is paramount [90].

### Spinal LITT

Patients with metastatic spinal tumors that cause epidural compression benefit from a combination of surgical decompression known as separation surgery followed by radiation therapy; in fact, Laufer et al. have demonstrated that separation surgery in combination with stereotactic radiation provides one-year local control rates of more than 91% regardless of tumor histology radiosensitivity [91]. Spinal LITT represents a novel minimally invasive approach to treating metastatic spine tumors. The surgical aim of LITT to treat spinal metastasis is to achieve local tumor control, allow for fast recovery, minimize postoperative pain and morbidity, and curtail delays in initiating or interrupting systemic therapies directed at the primary tumor [92]. The LITT technique has been introduced as an alternative to separation surgery and is used in a synergistic fashion with radiation therapy. The surgical aim of LITT to treat spinal metastasis is to achieve local tumor control, allow for fast recovery, minimize postoperative pain and morbidity, and curtail delays in initiating or interrupting systemic therapies directed at the primary tumor [92]. Spinal LITT is currently being done only at a small number of centers and most of the reports are retrospective.

Ahrar and Stafford first reported the use of LITT to treat spinal metastasis in 2010, but their study excluded tumors extending to the epidural space [93]. The authors concluded that LITT was a safe and reliable technique. More recently, Tatsui et al. found that LITT is safe and effective in patients with mild epidural compression secondary to tumor extension utilizing LITT as an option to replace separation surgery for specific patients as well as an adjunct to SSRS [94]. Specifically, patients go through LITT and then SSRS in standard doses to cover the gross tumor volume, as if no thermal ablation were performed. If spinal instability is suspected, percutaneous placement of spinal instrumentation and cement augmentation can be done in the same sitting, though this is not part of the LITT per se.

Details of operative considerations, placement of probe and ablation steps have been previously published in detail. Briefly, LITT procedures are done with an intraoperative MRI (iMRI) [90]. The diameter of the MRI bore must be large enough to fit the patient in the prone position with part of the probe protruding about 6 cm above the skin. The patient is positioned prone over gel rolls placed in parallel along the patient body axis with the arms tucked to the side. The probe is placed a distance of 6 mm from the ventral dural border of the posterior vertebral body using CT-based image guidance [95]. Tatsui et al. have found that a distance of 5 mm on each side of the laser fiber is covered by temperatures associated with tumor cell death [96]. If more coverage is needed, more than one probe may be used in various positions from different entry points in order to increase the area of cell death [95]. LITT is then performed. The heating process is monitored in real time with thermal MRI, and once the temperature reaches a critical level at the dural edge, the system deactivates, protecting the spinal cord from thermal damage. The LITT procedure is followed by SSRS in standard doses to cover the gross tumor volume. Patients undergoing LITT are admitted on the same day of the procedure with the average length of the procedure approaching eight hours. This time includes patient anesthesia and positioning, obtaining the fluoroscopic match for navigation, placing the access cannulas into the desired location in the epidural tumor, transferring the patient into the MRI magnet, obtaining the localization for each fiber, obtaining parameters for the thermal map for each fiber, performing an average of five cycles of heating per puncture with ventilator pauses, evaluating somatosensory evoked potential monitoring between each cycle, obtaining a final scan with and without contrast to evaluate the ablated tissue, closing the stab wounds, and transferring the patient to a stretcher for extubation. Post operatively, individuals are admitted to regular hospital beds after the procedure, and the median hospital stay was two days. Patients are discharged once pain is under control and they are typically capable of ambulating without assistance.

## Overall Conclusions

LITT is emerging as a safe, reliable, minimally invasive clinical approach, particularly for deep seated, focal malignant brain tumors and radiation necrosis. The role of LITT for treatment of other types of tumors of the brain and for spine tumors appears to be evolving at a small number of centers. While the technology appears to be safe and increasingly utilized, there have been few prospective clinical trials, and most published studies combine different pathologies in the same report. Well designed prospective trials for each of the various pathologies currently treated will be required to firmly establish the role of LITT in the treatment of lesions of the brain and spine.
